# Estimation of soil pH with geochemical indices in forest soils

**DOI:** 10.1371/journal.pone.0223764

**Published:** 2019-10-15

**Authors:** Wei Wu, Hong-Bin Liu

**Affiliations:** 1 College of Computer and Information Science, Southwest University, Beibei, Chongqing, China; 2 College of Resources and Environment, Southwest University, Beibei, Chongqing, China; Newcastle University, UNITED KINGDOM

## Abstract

Soil pH is a critical soil quality index and controls soil microbial activities, soil nutrient availability, and plant roots growth and development. The current study aims to evaluate various pedotransfer functions for predicting soil pH using different geochemical indices (CaO, ratios of Al_2_O_3_, Fe_2_O_3_, TiO_2_, SiO_2_, MgO, and K_2_O to CaO) in forest soils. Various models including empirical functions (quadratic, cubic, sigmoid, logarithmic) and artificial neural network with these geochemical indices were assessed by independent testing set. Mean bias error (MBE), root mean square error (RMSE), mean absolute percentage error (MAPE), mean absolute error (MAE), coefficient of determination (R^2^), t-statistics (t-stat), and Akaike’s Information Criterion (AIC) were applied to evaluate the model performances. Additionally, a new indicator (global performance indictor, GPI) was originally introduced in this study and was used to rank these models. According to GPI, the sigmoid functions and ANNs performed better than others. On average, they could explain above 70% of the variability in soil pH. Both model structure and dataset shape impact on model performance. The best input was CaO for ANNs, sigmoid, and logarithmic functions. The ratios of K_2_O to CaO and Al_2_O_3_ to CaO were the best inputs for quadratic and cubic equations, respectively.

## Introduction

Soil pH indicates soil acidity and alkalinity. Generally, slightly acidic soils are optimal for macro- and micro-nutrients availability [1]. Soil pH impacts on soil nutrients and plant growth and development [2]. It is a critical element for understanding soil nutrient availability and weathering as well as relationships between soil and biota. The relationship between soil pH and base saturation has been well studied. Some researchers observed a curvilinear relationship between soil pH and Ca saturation [3, 4]. Others reported a linear relationship between them [5, 6].

Soil CaO has been applied to predict soil pH with other geochemical elements. For example, Lukens et al. used ratios of Fe_2_O_3_, TiO_2_, and Al_2_O_3_ to CaO to predict soil pH with sigmoid functions [7]. The models produced similar prediction accuracy with coefficient of determination changing between 0.7 and 0.74, root mean square error between 0.83 and 0.88. Nordt and Driese found that bulk soil CaO + MgO could be used to predict soil pH in Vertisol [8]. The prediction of soil pH using bulk soil elemental oxides is also an issue in pedotransfer functions. Soil CaO, is one source of Ca^2+^ supply to soil solution, we believe that itself could be used to estimate soil pH. However, studies on this topic were limited.

The objectives of the current study were to (1) evaluate various pedotransfer functions for predicating soil pH using several geochemical indices and (2) investigate the usefulness of soil CaO to predict soil pH. To do this, five models with different geochemical indices were compared and tested. Specifically, artificial neural networks were evaluated with respect to the non-linear relationship between soil pH and the geochemical indices. Model performances were evaluated by an independent validation set.

## Materials and methods

### Study site

**Fig 1:**
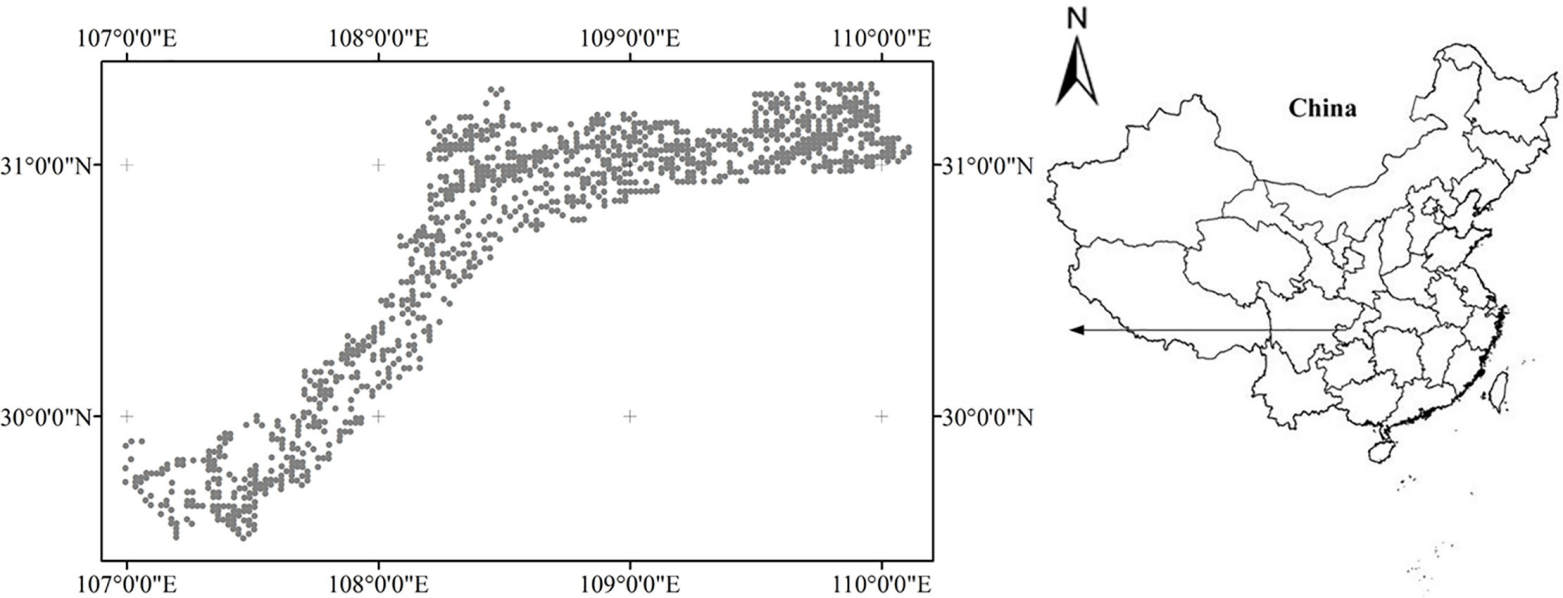
Maps of study area location and sample sites.

The study area covering 13326 km^2^ is located in the core region of the Three Gorges Reservoir of China (Fig 1). It has a humid subtropical monsoon climate with a mean annual precipitation of 1267 mm and a mean annual temperature of 16.02°C. The elevation varies between 175 and 2033 m with a mean of 643 m. The slope changes between 0.45° and 52.96° with a mean of 17.83°.

### Data

A total of 1163 samples were collected from forest soils in the study area (Fig 1), where the major bedrock lithologies are carbonate rocks and sandstone and soil type is Combisols [9]. The study did not involve private land, protected land, endangered or protected species. No specific permissions were required for these locations/activities. In order to ensure an even distribution of selected sites, systematic sampling using a regular grid was applied in this work [10]. Surface soils at 0–20 cm depth were collected at a density of 1 sample/km^2^. For each sampling site, 3 to 5 subsamples collected within 50 m of the site were mixed to represent the sample. All the sampling locations were recorded by Global Positioning System (GPS). Standard measurements were performed on the soil samples. Prior to laboratory analysis, samples were air-dried and passed through a 2 mm soil sieve. Soil pH was determined in a soil-to-water ratio of 1:2.5 with a glass electrode. The elements (Al_2_O_3_, Fe_2_O_3_, TiO_2_, SiO_2_, K_2_O, Mg_2_O, and CaO) were measured by Inductively Coupled Plasma-Optical Emission Spectrometry (ICP-OES) method [10].

Ratios of Al_2_O_3_, Fe_2_O_3_, TiO_2_, SiO_2_, MgO, and K_2_O to CaO (hereafter AlCa, FeCa, TiCa, SiCa, MgCa, and KCa) and CaO were used to develop the pedotransfer functions to predict soil pH in forest soils [7]. These geochemical indices were calculated by

$$G=\frac{X}{X+CaO}\times 100$$ (1)

where X represents Al_2_O_3_, Fe_2_O_3_, TiO_2_, SiO_2_, MgO, and K_2_O.

All data were divided into calibration and validation sets for each dataset. Approximately 2/3 of the data were used to develop (or train) the models. The remaining 1/3 of the data were used to validate the models.

### Models

Both empirical functions (quadratic, cubic, sigmoid, and logarithmic) and artificial neural network were tested in this work. The expressions of these empirical functions are given in Table 1. For sigmoid function, parameter k and p are the minimum and range of the response, respectively.

**Table 1:** Empirical models used in the current study.

Name	Ab.	Equation
**Quadratic**	Q	y = *b*_0_+*b*_1_x+*b*_2_x^2^
**Cubic**	C	y = *b*_0_+*b*_1_x+*b*_2_x^2^+*b*_3_x^3^
**Sigmoid**^**a**^	Sig	$y=k+\frac{p}{1+{\left(\frac{x}{b_{0}}\right)}^{{-b}_{1}}}$
**Logarithmic**	Log	y = *b*_0_+*b*_1_ln(*x*)

^a^k and p are the minimum and range of the response, respectively.

**Fig 2:**
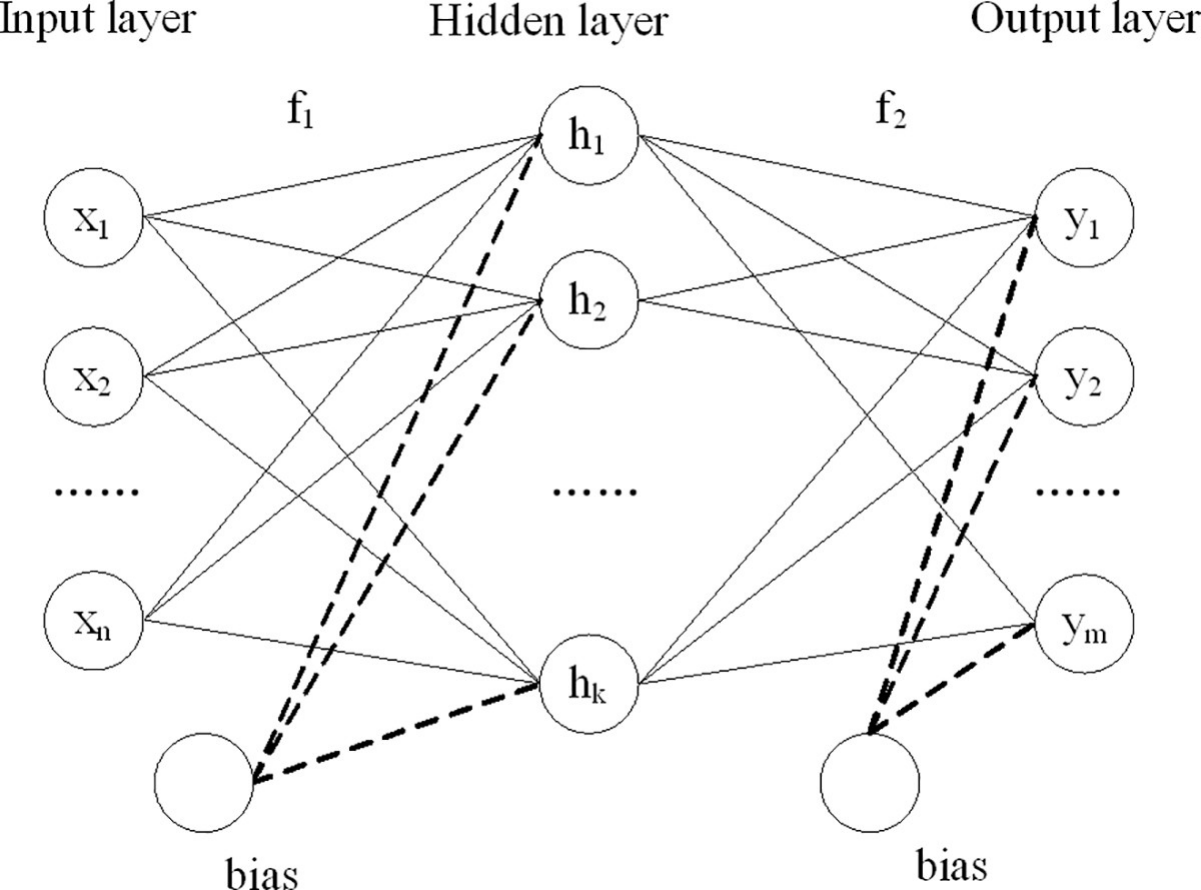
ANN structure.

The artificial neural networks (ANNs) that are inspired by biological neural network are also frequently used tools for various fields [11–13]. ANNs can deal with both linear and non-linear relationships between variables [11, 12]. In the current study, ANNs with three layers (an input, a hidden, and an output layers) were tested and trained with scale conjugate gradient back propagation algorithm (Fig 2). The output of a node is,

$$y_{j}=f(\sum_{i=1}^{n}x_{i}∙w_{ij}-b_{j})$$ (2)

where *f* is an activation function, *y* is the output of a node *j*, *x*_*i*_ is an input of the vector of inputs, *w*_*ij*_ is the weight connected the input *x*_*i*_ to the node *j*, and *b*_*j*_ is a bias associated with the node *j*. The parameters (weight and bias) are determined during the training stage based on a set of input data and targets. The tangent and linear activation functions were used in the hidden layer and output layer, respectively [14–17].

The numbers of neurons in the hidden layer between 2 and 20 were tried. To train the ANNs, three datasets were created randomly based on the calibration dataset for training (70%), validating (15%), and testing (15%). The ANNs with the lowest value of root mean square error (RMSE) and the highest value of coefficient of determination (R^2^) were selected to predict soil pH using the geochemical indices. Number of parameters was calculated by [18],

$$N=(N_{i}+1)\times N_{h}+(N_{h}+1)\times N_{o}$$ (3)

where *N*_*i*_, *N*_*h*_, *N*_*o*_, and 1 are number of node in the input, hidden, output layers and bias, respectively.

### Performance evaluation

Model performances could be evaluated by comparing predicted and measured data based on a set of statistical error indicators. In this work, mean bias error (MBE), root mean square error (RMSE), mean absolute percentage error (MAPE), mean absolute error (MAE), and coefficient of determination (R^2^), t-statistics (t-stat), and Akaike’s Information Criterion (AIC) [19] were employed to assess the model performances based on the independent validation set.

$$MBE=\frac{1}{n}\sum_{i=1}^{n}\left(y_{i}-{\hat{y}}_{i}\right)$$ (4)

$$RMSE=\sqrt{\frac{\sum_{i=1}^{n}{\left(y_{i}-{\hat{y}}_{i}\right)}^{2}}{n}}$$ (5)

$$MAPE=\frac{1}{n}\sum_{i=1}^{n}\left|\frac{y_{i}-{\hat{y}}_{i}}{y_{i}}\right|$$ (6)

$$MAE=\frac{1}{n}\sum_{i=1}^{n}\left|y_{i}-{\hat{y}}_{i}\right|$$ (7)

$$R^{2}=1-\frac{\sum_{i=1}^{n}{\left(y_{i}-\bar{y}\right)}^{2}}{\sum_{i=1}^{n}{\left(y_{i}-\hat{y_{i}}\right)}^{2}}$$ (8)

$$t-stat=\sqrt{\frac{(n-1){MBE}^{2}}{{RMSE}^{2}-{MBE}^{2}}}$$ (9)

$$AIC=ln\left(\frac{\sum_{i=1}^{n}\left(y_{i}-{\hat{y}}_{i}\right)}{n}\right)+\frac{2(k+1)}{n}$$ (10)

where n is the number of observations, *y*_*i*_, and *ŷ*_*i*_ are the measured and estimated soil pH of the ith soil sample, respectively, $\bar{y}$ is the mean value of the measured soil pH, k is the number of parameters. MBE shows overall under- or over-estimation tendency. A negative value of MBE indicates an overestimation of the model, and a positive one indicates an underestimation of the model. The most accurate model has an MBE value closed to zero, lower values of RMSE, MAPE, MAE, t-stat, AIC, and a higher value of R^2^.

Each statistical error indicator has its specific strength and weakness. For example, RMSE is not a better indicator than MBE for evaluating average model performance [20]. However, MBE could not give the correct performance when the model has overestimations and underestimations at the same time. Therefore, to find out the best model based on the above-mentioned indicators, a new Global Performance Indicator (GPI) was introduced in this work. Each indicator should be scaled on a scale of 0–1 with 0 being the best and 1 representing the worst. For the indicators that have negative or positive values, their absolute values are used in GPI. For the indicators that the lower the better (e.g., RMSE and MAPE etc.), the minimum is scaled to 0 and maximum to 1 (Eq 11). For the indicators that the higher the better (e.g., R^2^), the maximum is scaled to 0 and minimum to 1 (Eq 12). For the ith model, the GPI was defined as,

$$I=\frac{P-P_{min}}{P_{max}-P_{min}}$$ (11)

$$I=\frac{P_{max}-P}{P_{max}-P_{min}}$$ (12)

$${GPI}_{i}=\sum_{j=1}^{m}I_{ij}$$ (13)

Where P is the performance indicator. P_max_ and P_min_ are the maximum and minimum of P for the corresponding indicators of the evaluated models. I_ij_ is the scaled value of indicator j for the *ith* model and *m* is the number of performance indicators. Models with GPI closer to zero perform better.

### Statistical analysis

A one-way analysis of variance (ANOVA) was used to test the difference in variables between calibration and validation sets. Pearson’s correlation coefficients were calculated to determine the strength of correlations between soil pH and geochemical indices. The analyses of descriptive statistics were performed in SPSS v13.0. Model development and validation were done by MATLAB v9.0.

## Results

### Data overview

**Fig 3:**
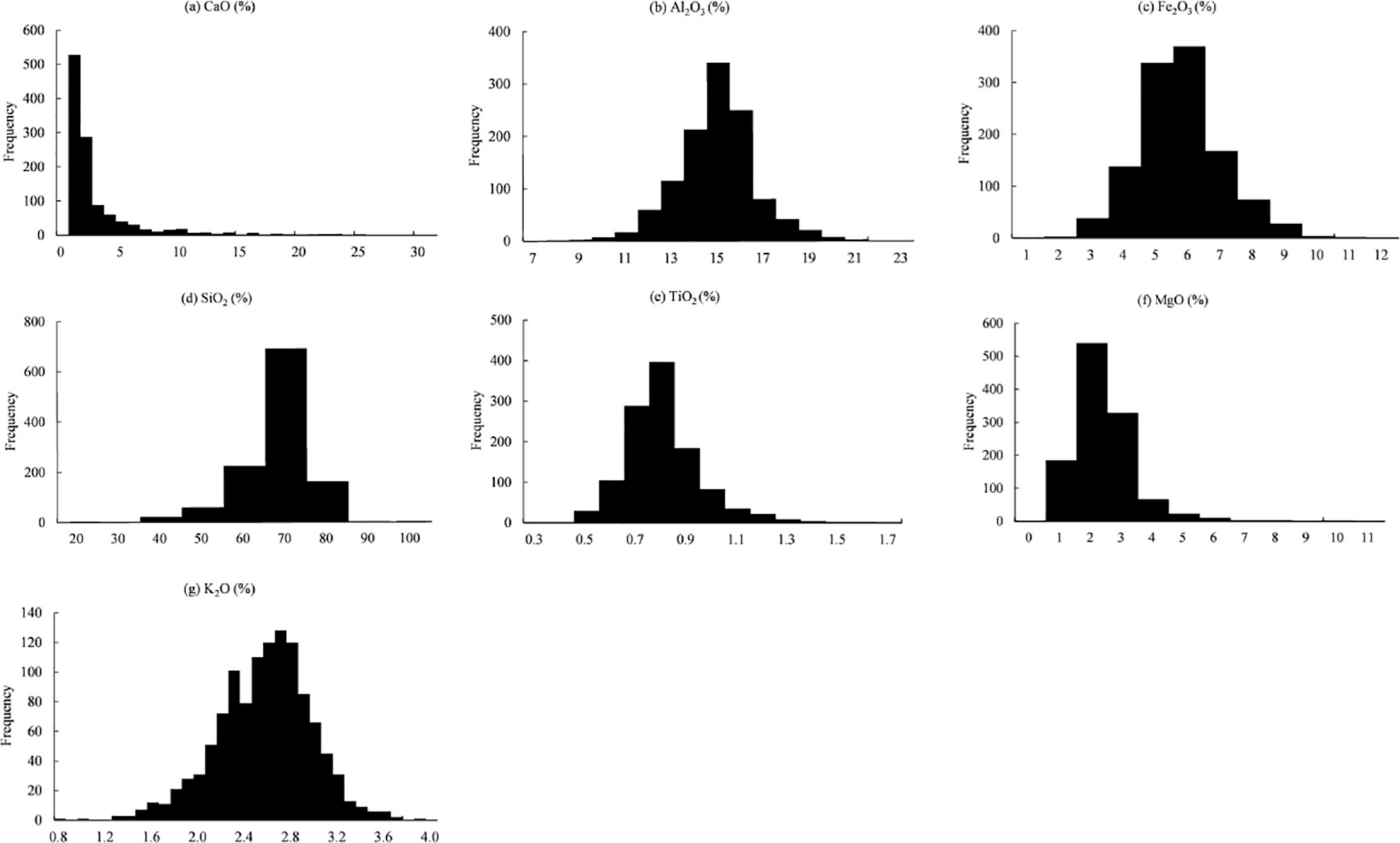
Histogram plots for the geochemical elements.

On average, the soils were neutral. Soil pH varies between 4.34 and 8.7 with a mean of 7.16 (Table 2). CaO mainly ranged between 0 and 30% (mean = 2.63%), Al_2_O_3_ between 12 and 15% (mean = 14.4%), Fe_2_O_3_ between 3 and 6% (mean = 5.2%), TiO_2_ between 0.5 and 0.8% (mean = 0.75%), SiO_2_ between 50 and 70% (mean = 62.9%), MgO between 0 and 2% (mean = 1.9%), K_2_O between 2.2 and 2.7% (mean = 2.5%) (Fig 3). In terms of coefficient of variation (CV%), soil pH showed low variability (< 25%). Among the geochemical indices, SiCa and AlCa presented low variability (< 25%), FeCa, TiCa, MgCa, KCa showed medium variability (25% - 75%) and CaO presented high variability (> 75%).

**Table 2:** Descriptive statistics of soil pH and geochemical indices (N = 1163).

	Min	Max	Median	Mean	Std. Dev	CV%
**pH**	4.34	8.7	7.46	7.16	1.09	15.22
**CaO (%)**	0.08	29.98	1.10	2.63	4.05	153.77
**AlCa (%)**	24.62	99.54	93.09	87.55	13.59	15.52
**FeCa (%)**	10.09	98.53	82.67	75.48	19.05	25.24
**TiCa (%)**	1.35	91.00	39.71	39.57	21.32	53.86
**SiCa (%)**	48.65	99.87	98.31	95.71	6.93	7.24
**MgCa (%)**	4.46	92.26	61.57	55.87	17.23	30.84
**KCa (%)**	5.73	95.99	70.33	63.4	21.35	33.67

**Fig 4:**
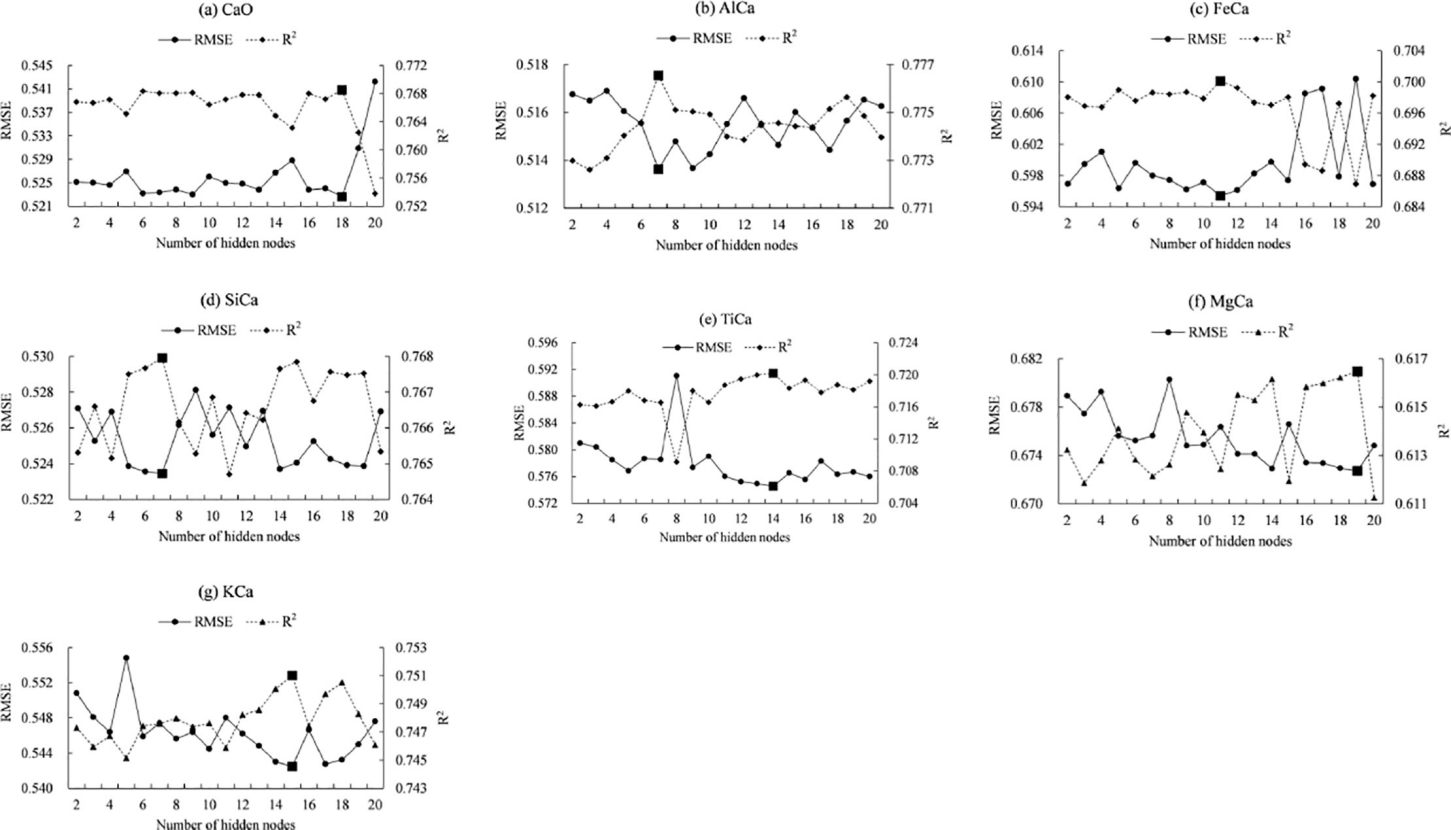
Relationships between soil pH and the geochemical indices.

Soil pH showed significant correlation with these geochemical indices (Table 3 and Fig 4).

**Table 3:** Pearson’s correlation coefficients between soil pH and geochemical indices (p<0.01).

CaO (%)	AlCa (%)	FeCa (%)	TiCa (%)	SiCa (%)	MgCa (%)	KCa (%)
0.5	-0.61	-0.68	-0.83	-0.49	-0.71	-0.76

Differences in soil pH and geochemical indices between calibration and validation sets were given in Table 4. Results of ANOVA indicated that there was no significant difference in these variables between calibration and validation sets.

**Table 4:** Differences in soil pH and geochemical indices between calibration and validation sets (N = 877 and 286 for calibration (Cal) and validation (Val) sets, respectively.).

Item		Min	Max	Median	Mean	Std.Dev	F	p value
**pH**	Cal	4.52	8.6	7.41	7.14	1.09	0.954	0.329
	Val	4.34	8.7	7.57	7.21	1.08		
**CaO(%)**	Cal	0.11	29.98	1.10	2.62	4.04	0.014	0.904
	Val	0.08	24.74	1.11	2.66	4.08		
**AlCa(%)**	Cal	24.62	99.03	93.18	87.57	13.67	0.008	0.929
	Val	31.14	99.54	93.07	87.48	13.38		
**FeCa(%)**	Cal	10.09	97.46	82.67	75.55	19.12	0.048	0.827
	Val	13.79	98.53	82.67	75.26	18.85		
**TiCa(%)**	Cal	1.35	85.70	39.71	39.65	21.25	0.050	0.823
	Val	1.82	91.00	39.7	39.33	21.54		
**SiCa(%)**	Cal	48.65	99.87	98.31	95.72	6.90	0.015	0.902
	Val	57.80	99.87	98.3	95.66	7.00		
**MgCa(%)**	Cal	4.46	91.72	61.73	56.03	0.58	0.284	0.594
	Val	6.83	92.26	61.07	55.4	1.05		
**KCa(%)**	Cal	5.73	94.31	70.2	63.52	0.72	0.105	0.746
	Val	7.08	95.99	70.66	63.04	1.27		

### Model calibration

**Fig 5:**
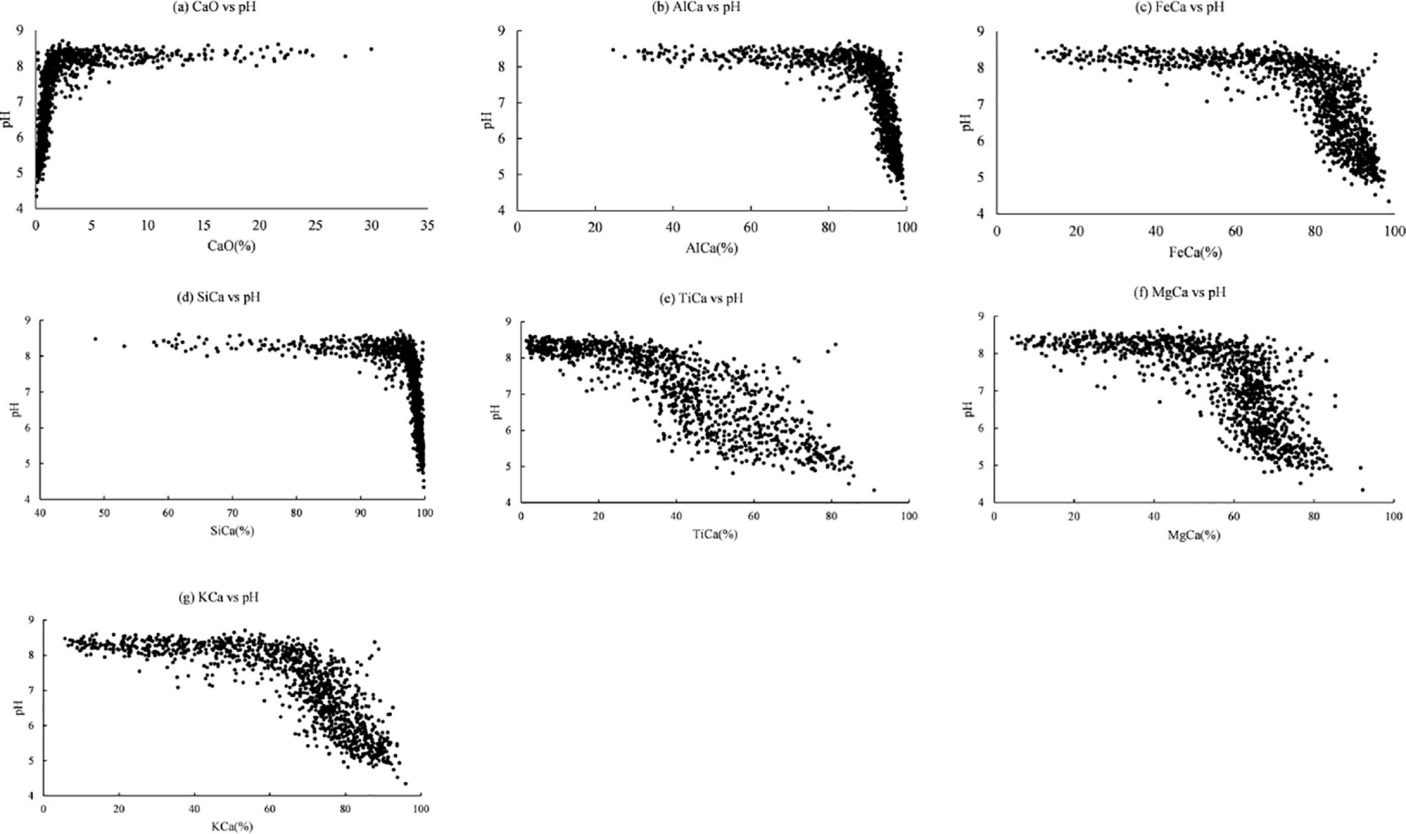
Root mean square error (RMSE) and coefficient of determination (R2) for ANNs with different numbers of hidden nodes (The black box indicates the lowest value of RMSE or highest value of R^2^).

The coefficients of determination (R^2^) of the developed models based on the calibration set are given in Table 5. The ANNs with 18, 7, 11, 7, 14, 19, and 15 hidden nodes were applied to estimate soil pH using CaO, AlCa, FeCa, SiCa, TiCa, MgCa, KCa, and respectively (Fig 5). On average, ANN produced the highest value of R^2^ (0.73), followed by sigmoid (R^2^ = 0.7) and cubic (R^2^ = 0.63) equations. The values of R^2^ ranged between 0.21 (p < 0.01, logarithmic equation with SiCa) and 0.77 (p < 0.01, ANN with SiCa).

**Table 5:** Model calibration (N = 877, p<0.01).

Input	Function	b0	b1	b2	b3	R^2^
**CaO**	Quadratic	6.4103	0.4148	-0.0156		0.43
	Cubic	6.0427	0.8099	-0.0684	0.0016	0.56
	Sigmoid	0.6823	1.3914			0.74
	Logarithmic	6.9224	0.7888			0.64
	ANN					0.76
**AlCa**	Quadratic	1.281	0.2493	-0.002		0.59
	Cubic	24.0871	-0.8736	0.0151	-0.00008	0.71
	Sigmoid	94.9733	31.04			0.73
	Logarithmic	20.2979	-2.9535			0.3
	ANN					0.77
**FeCa**	Quadratic	6.3399	0.1023	-0.0011		0.65
	Cubic	9.7379	-0.1244	0.0031	-0.00002	0.69
	Sigmoid	87.0113	10.6616			0.67
	Logarithmic	14.689	-1.7664			0.34
	ANN					0.7
**TiCa**	Quadratic	8.6033	-0.0276	-0.00018		0.69
	Cubic	8.2332	0.02226	-0.00167	0.000012	0.7
	Sigmoid	51.1643	2.465			0.7
	Logarithmic	10.4478	-0.9595			0.51
	ANN					0.72
**SiCa**	Quadratic	-22.1396	0.8114	-0.0053		0.42
	Cubic	-2.4129	0	0.0055	-0.000046	0.45
	Sigmoid	98.8379	120.5882			0.73
	Logarithmic	34.5359	-6.0105			0.21
	ANN					0.77^a^
**MgCa**	Quadratic	7.6946	0.0516	-0.001		0.6
	Cubic	7.4604	0.0718	-0.0015	3.352E-6	0.6
	Sigmoid	67.2264	5.7029			0.6
	Logarithmic	13.2347	-1.5416			0.37
	ANN					0.61
**KCa**	Quadratic	7.4643	0.0595	-0.0009		0.73
	Cubic	8.1355	0.0045	0.0003	-7.469E-6	0.73
	Sigmoid	77.0693	7.1629			0.73
	Logarithmic	12.8597	-1.4093			0.41
	ANN					0.76

^a^Box in grey denoted the highest value of R^2^.

### Model performance

Performances of the models were evaluated based on the validation set and the statistical error indicators were shown in Table 6. On average, all models except sigmoid functions presented underestimation tendency according to MBE. In terms of MAPE, models gave good estimation of soil pH (mean MAPE = 7.4%). ANN and sigmoid models could explain above 70% of the variability in soil pH (R^2^ = 0.73 and 0.71, respectively). Logarithmic model performed worst with the highest values of MBE, RMSE, MAPE, MAE, AIC, and the lowest values of R^2^. ANN gave the best estimations of soil pH according to RMSE, MAPE, MAE, t-stat, and R^2^. Sigmoid model performed best based on AIC and MBE. The geochemical indices gave varied prediction performances with models. For example, SiCa produced the highest R^2^ in ANNs, KCa in quadratic and cubic functions, CaO in logarithmic and sigmoid models. Lukens et al. [7] predicted soil pH by AlCa, FeCa, and TiCa using sigmoid models. They reported that TiCa and FeCa gave slightly better performances than AlCa. In the current work, CaO, AlCa, SiCa, and KCa produced better predictions of soil pH than FeCa and TiCa using sigmoid functions based on R^2^.

**Table 6:** Model performance (N = 286).

Fun.	Input	MBE	RMSE	MAPE	MAE	R^2^	AIC	t-stat	GPI	Rank
**ANN**	AlCa	0.014	0.514	0.054	0.371	0.78	-0.446	0.472	1.01	2
	FeCa	0.007	0.587	0.064	0.439	0.71	-0.226	0.207	2.84	5
	SiCa	0.024	0.508	0.054	0.365	0.78	-0.455	0.794	1.21	3
	TiCa	0.039	0.565	0.061	0.414	0.73	-0.195	1.165	3.3	6
	MgCa	0.034	0.679	0.075	0.507	0.61	-0.362	0.847	5.52	7
	KCa	0.069	0.534	0.057	0.393	0.76	-0.925	2.218	2.6	4
	CaO	0.014	0.512	0.054	0.367	0.78	-0.557	0.476	0.81	1
**Q**	AlCa	0.047	0.698	0.085	0.589	0.59	-0.692	1.132	3.08	4
	FeCa	0.04	0.638	0.074	0.514	0.65	-0.87	1.059	1.55	2
	SiCa	0.068	0.825	0.104	0.71	0.42	-0.356	1.389	6.48	7
	TiCa	0.061	0.591	0.065	0.446	0.71	-1.024	1.743	2.33	3
	MgCa	0.058	0.694	0.077	0.527	0.5	-0.702	1.412	3.72	5
	KCa	0.052	0.556	0.061	0.42	0.74	-1.147	1.579	1.19	1
	CaO	0.066	0.815	0.102	0.7	0.44	-0.389	1.377	6.19	6
**C**	AlCa	0.036	0.579	0.067	0.465	0.72	-1.056	1.043	1.21	1
	FeCa	0.046	0.595	0.065	0.447	0.7	-1.004	1.308	2.23	3
	SiCa	0.051	0.721	0.089	0.61	0.56	-0.619	1.206	6	7
	TiCa	0.051	0.584	0.063	0.431	0.71	-1.04	1.484	2.42	4
	MgCa	0.056	0.694	0.077	0.529	0.59	-0.695	1.37	5.26	5
	KCa	0.054	0.543	0.058	0.403	0.75	-1.185	1.689	1.9	2
	CaO	0.048	0.71	0.088	0.601	0.57	-0.658	1.151	5.49	6
**Log**	AlCa	0.072	0.919	0.117	0.797	0.28	-0.148	1.326	5.88	6
	FeCa	0.07	0.885	0.112	0.765	0.34	-0.223	1.343	5.33	5
	SiCa	0.068	0.976	0.125	0.85	0.19	-0.027	1.179	6.32	7
	TiCa	0.063	0.769	0.095	0.654	0.5	-0.505	1.4	3.43	3
	MgCa	0.046	0.861	0.108	0.736	0.61	-0.278	0.903	2.75	2
	KCa	0.059	0.845	0.106	0.726	0.4	-0.317	1.175	4.01	4
	CaO	0.056	0.642	0.076	0.53	0.65	-0.873	1.486	1.38	1
**Sig**	AlCa	-0.065	0.557	0.063	0.435	0.75	-1.135	1.988	2.68	4
	FeCa	-0.043	0.625	0.072	0.494	0.68	-0.904	1.161	4.06	6
	SiCa	-0.072	0.55	0.062	0.429	0.76	-1.159	2.239	2.69	5
	TiCa	0.003	0.593	0.066	0.453	0.7	-1.012	0.085	1.89	3
	MgCa	-0.005	0.701	0.079	0.541	0.59	-0.677	0.125	5.05	7
	KCa	-0.027	0.564	0.062	0.429	0.74	-1.11	0.8	1.67	2
	CaO	0.033	0.533	0.057	0.397	0.76	-1.231	1.045	0.88	1
**Mean**	Q	0.056	0.688	0.081	0.558	0.58	-0.740	1.384	4.16	4
	Cubic	0.049	0.632	0.072	0.498	0.66	-0.894	1.322	2.79	3
	Log	0.062	0.842	0.106	0.723	0.42	-0.339	1.259	6.75	5
	Sig	-0.025	0.589	0.066	0.454	0.71	-1.033	1.063	0.82	1
	ANN	0.029	0.557	0.060	0.408	0.73	-0.452	0.883	0.93	2
Overall mean		0.034	0.629	0.074	0.507	0.59	-0.566	1.184		

Box in grey presented the best performance suggested by the corresponding error indicator.

Models gave different prediction accuracy indicated by different statistical error indicators. For example, ANN with SiCa was the best one in terms of RMSE, MAPE, MAE, and R^2^. Sigmoid function with TiCa performed best based on MBE and t-stat. Cubic with KCa was the best according to AIC.

**Fig 6:**
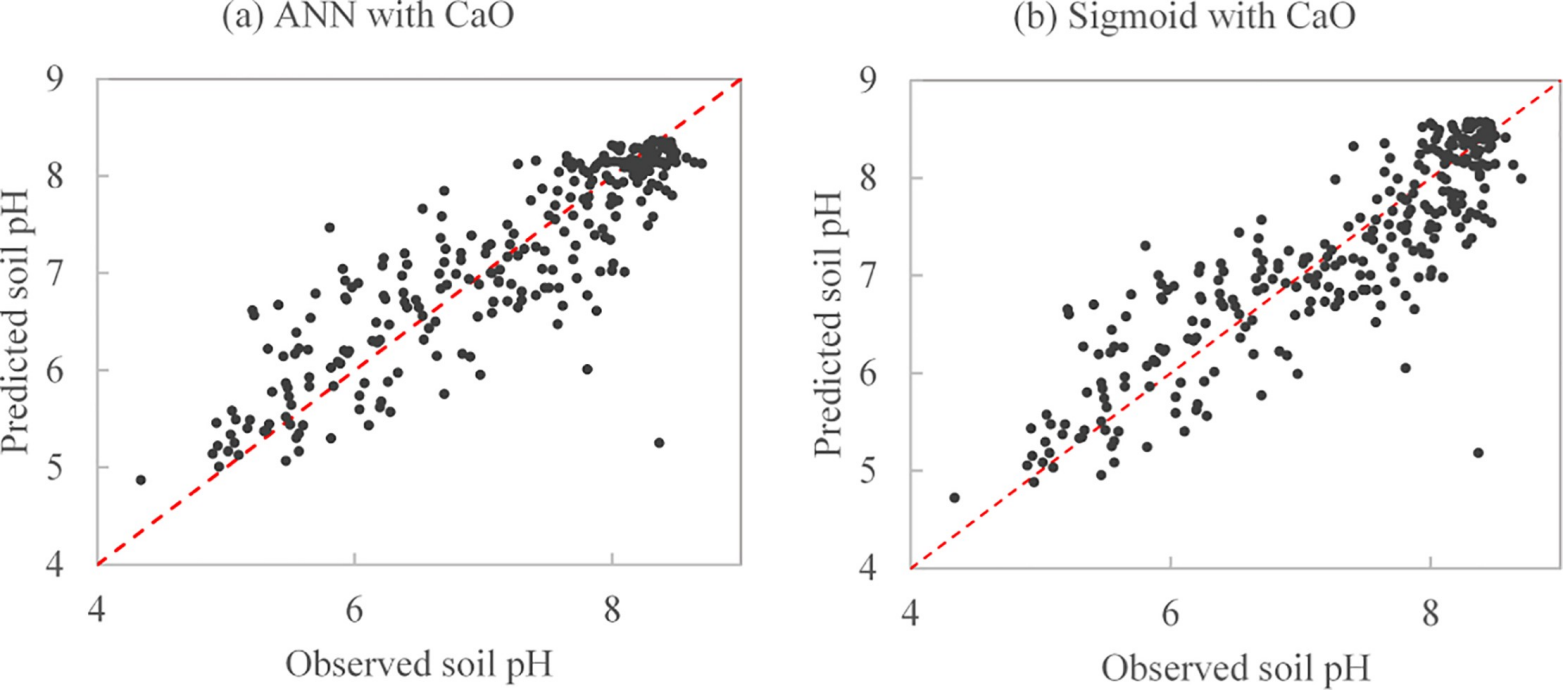
Scatter plot of the observed and predicted soil pH by (a) artificial neural network with CaO and (b) sigmoid with CaO. The red dash line is the 1:1 line.

Because the used statistical error indicators did not always give the consistent results, the GPI was introduced and calculated by combining these indicators. The ranking of the models according to each accuracy indicator and GPI was reported in Table 6. On average, the results of GPI indicated that sigmoid model, ANN, and cubic were ranked 1st, 2nd, and 3rd. The model performance indicated by GPI was acceptable and better, because it combined all the performance tests. GPIs were also calculated within each model. The geochemical indices gave different performance for the evaluated models. CaO ranked 1st in ANNs, sigmoid and logarithmic functions. KCa ranked 1st in quadratic models. Therefore, CaO and KCa were the best inputs to predict soil pH for both ANNs and the empirical equations over the study site. Scatter plots of the observed and predicted soil pH by ANN with CaO and sigmoid with CaO were given in Fig 6. Statistics of validation results were listed in Table 7. The maximum pH values were underestimated while the minimums were overestimated for both models. There was no significant difference in soil pH between observations and predictions for the two models.

**Table 7:** Statistics of validation results (N = 286).

pH	Min	Max	Median	Mean	Std.Dev	F	p value
**Observation**	4.34	8.7	7.57	7.21	1.08		
**Predicted by ANN with CaO**	4.87	8.36	7.35	7.2	0.98	0.028	0.868
**Predicted by sigmoid with CaO**	4.72	8.57	7.22	7.18	1	0.143	0.706

## Discussion

On average, ANNs performed better than cubic, quadratic, and logarithmic functions. Among the empirical approaches, sigmoid function was the best one. Model structure results in the differences between them [21]. ANN constructs a network connected with weighted nodes that were trained by certain algorithms. Compared with other models, the main advantages of ANNs are: 1) they are non-parametric techniques and do not need any model assumptions; 2) ANNs have no assumption on data distribution. Generally, ANN is often criticized for its complex network structure that makes the results difficult to interpret [22]. The indicator, AIC, based on an “information-theoretical approach” has been widely used for model selection [23–25]. In this case, ANNs produced higher values of AIC than others, due to the larger number of model parameters. Besides, data set shape also impacts on model performance, especially for the empirical functions. The rank order of them are sigmoid > cubic > quadratic > logarithmic functions. The best input was CaO for ANNs, sigmoid and logarithmic functions. The ratios of K_2_O to CaO and Al_2_O_3_ to CaO were the best inputs for quadratic and cubic equations, respectively.

CaO and the ratios of elemental oxides to CaO could be used to predict soil pH, because Ca^2+^ is the main driver affecting soil pH [7]. The sigmoid functions indicated the geochemical indices have different rates of change in soil pH. This was also given by the scatter plots (Fig 4). The oxides that were more abundant than CaO had higher values of growth rate and inflection point (e.g., SiO_2_, Al_2_O_3_, Fe_2_O_3_) and vice versa (e.g., TiO_2_, MgO, K_2_O). Lukens et al. (2018) stated that samples collected from calcareous soils could have a relatively large values of FeCa or AlCa and compressed intervals at higher index values, where pH decreases as a function of Ca loss and Fe or Al gain. This could also explain the relationships between soil pH and the ratios of elemental oxides to CaO over the current study site.

Soil pH is a key parameter for understanding soil weathering and relationships between soil nutrient availability and environmental factors. Weathering indices that incorporate Ca in some form could track soil pH. A recent study reported that soil pH values are closely correlated with water balance (mean annual precipitation–mean annual potential evapotranspiration) at global scale [26]. The pedotransfer functions and geochemical proxies compared and evaluated in the current study could be used to estimate significantly environmental components in the past time [7].

## Conclusions

Various pedotransfer functions with different geochemical indices were applied to estimate soil pH in forest soils. The predicted data were compared to the measurements of an individual validation dataset. In order to do so, 7 statistical indicators have been applied to test models performances. Moreover, a new accuracy factor, named Global Performance Indicator (GPI), was originally introduced in this study and was used to rank the proposed models. The rank order was sigmoid > artificial neural network > cubic > quadratic > logarithmic. Soil CaO could be used to predict soil pH with ANNs, sigmoid and logarithmic functions. KCa and AlCa were the best inputs for quadratic and cubic equations, respectively.

## Supporting information

S1 File Data. (CSV)
